# Molecular Profiling of Soil-Derived *Bacillus subtilis* and *Bacillus paralicheniformis*: Evaluation of Anticancer and Antibacterial Efficacy Against Gastrointestinal Pathogens

**DOI:** 10.3390/cimb48050514

**Published:** 2026-05-15

**Authors:** Rida-e-Zainab Kayani, Komal Aman, Tajamul Hussain, Hazir Rahman, Ziaur Rahman, Ahmed Muhammad Ajaz, Muhammad Latif, Salman Alrokayan

**Affiliations:** 1 Department of Microbiology, Abdul Wali Khan University, Mardan 23200, Khyber Pakhtunkhwa, Pakistan; ridakayani04@gmail.com (R.-e.-Z.K.); hazirrahman@awkum.edu.pk (H.R.); 2 Department of Microbiology, Women University Mardan, Mardan 23200, Khyber Pakhtunkhwa, Pakistan; dr.komalaman@wumardan.edu.pk; 3 Center of Excellence in Biotechnology Research, King Saud University, Riyadh 11451, Saudi Arabia; thussain@ksu.edu.sa; 4 Institute of Green-Bio Science and Technology, Seoul National University, Pyeongchang-gun 232-916, Gangwon-do, Republic of Korea; m.ajaz@snu.ac.kr; 5 Department of Chemistry, Rawalpindi Women University (RWU), Rawalpindi 43600, Punjab, Pakistan; 6 Centre for Genetics and Inherited Diseases (CGID), Taibah University, Madinah 42353, Saudi Arabia; 7 Department of Basic Medical Sciences, College of Medicine, Taibah University, Madinah 42353, Saudi Arabia; 8 Biochemistry Department, College of Science, King Saud University, Riyadh 11451, Saudi Arabia; salrokayan@ksu.edu.sa

**Keywords:** molecular profiling, soil bacterial isolates, metabolic profiles, anticancer activity, antibacterial activity

## Abstract

***Background*****:** Microbial derivatives are pivotal in research because of their structural heterogeneity and diverse biological significance, including antitumor and antimicrobial activities. ***Aims and Objectives*****:** This study aimed to evaluate the molecular characterization, antibacterial effects on multidrug-resistant (MDR) gastrointestinal pathogens, and potential anticancer activity of soil bacterial isolates. ***Methodology*****:** Two bacterial isolates, *Bacillus subtilis* (*B. subtilis*) and *Bacillus paralicheniformis* (*B. paralicheniformis*), were characterized for their metabolic profiles and antimicrobial efficacy. Evolutionary relationships were determined using Sanger sequencing and MEGA-7 software. Secondary metabolites were characterized using Gas Chromatography–Mass Spectrometry (GC-MS), potentially contributing to the development of novel therapeutic agents. ***Results*****:** *B. subtilis* and *B. paralicheniformis* were identified as microbial strains, with 11 and 28 compounds isolated from their extracts, respectively, and evaluated for their efficacy against gastrointestinal pathogens. The extracts also demonstrated potential anticancer effects, with significant DPPH-scavenging activity. *B. subtilis* exhibited higher Superoxide Dismutase (SOD) activity, suggesting the presence of antioxidant bioactive compounds. This study also documented alterations in the apoptotic and anti-apoptotic gene expression patterns in HCT-116 cells treated with the extracts. Notably, the increase in *BAK* and *BAX* expression suggests a possible link to the apoptotic response in HCT-116 cells. ***Conclusions*****:** This study highlights the diversity of bacterial communities in soil that produce numerous secondary metabolites with therapeutic potential, including antibacterial and anticancer properties.

## 1. Introduction

Cancer is a global non-communicable health catastrophe. In 2024, approximately 10 million cancer-related mortalities and 19.3 million new cases were reported worldwide. While various synthetic molecules have been developed, compounds synthesized by bacterial species have attracted considerable interest in the development of novel therapeutic modalities for a wide range of human diseases, including oncological disorders [1]. Compounds derived from prokaryotic organisms have progressively attracted attention for the discovery of novel therapeutic agents for a myriad of human ailments, including gastrointestinal, respiratory, and urinary tract infections, as well as several animal diseases [2]. It is imperative to focus on exploring less toxic and more efficacious antibiotics derived from non-pathogenic microorganisms to address the burgeoning issue of multidrug resistance (MDR), particularly in comparison to existing antimicrobial agents. For instance, antibiotics used for the treatment of patients are mostly derived from bacterial sources, such as *Streptomyces* spp. [3].

Microorganisms synthesize a diverse array of key compounds that enhance their survival and competitive advantage in their ecological niches. Among these metabolites, several have substantial applications in biotechnology and medicine, notably as antibiotics. Historically, most antibiotics have been extracted from soil-dwelling bacteria and subsequently cultured under laboratory conditions [4]. Microbial derivatives have proven indispensable in research owing to their structural heterogeneity and diverse biological significance, including antitumor and antimicrobial activities [5]. These include antitumor agents, cholesterol-lowering drugs, and antibiotics [6]. Contemporary research has focused on the isolation and assessment of microbial compounds under various natural conditions involving microorganisms. The emergence of MDR represents a critical concern, contributing to elevated mortality rates, protracted illness, and considerable financial burden on healthcare systems and patients. The United Nations General Assembly has underscored this issue as a prominent health threat, necessitating immediate intervention [7].

Since the introduction of antibiotics in medical practice, healthcare systems have encountered substantial challenges owing to the increasing prevalence of drug-resistant and MDR bacterial strains. This issue encompasses various sectors, including the political, economic, biological, social, and ecological domains, with uncertain outcomes and no immediate solutions. The prolonged use of antimicrobials has facilitated the evolution of bacterial resistance, thereby reducing the ability to manage key diseases and leading to economic losses and adverse impacts on public health. Over the past two decades, bacterial resistance has evolved rapidly and is now acknowledged as a critical global public health concern, resulting in increased infections, prolonged symptoms, extended hospitalizations, and rising healthcare costs [8,9]. Resistance genes in bacterial populations have emerged through a multitude of mechanisms, encompassing both intrinsic and acquired resistance, as well as horizontal and vertical gene transfer modalities such as conjugation, transduction, and transformation [10]. The primary challenges related to MDR arise from the development of pathogenic resistance in conjunction with a reduction in the discovery of new bioactive compounds. Moreover, there is an increasing demand for environmentally friendly compounds with a reduced ecological impact. Microorganisms can interact in different ways, inhibiting their growth through various biochemical processes [11]. Therefore, advancements in the development of laboratory-synthesized antibiotics have saved millions of lives; however, the sustained discovery of new antimicrobial agents presents significant challenges. This predicament is partly attributable to the evolution of resistance among microorganisms, particularly Gram-negative *bacilli* such as *Pseudomonas aeruginosa*, *Klebsiella* spp., *Escherichia coli*, *Acinetobacter* spp., and *Salmonella* spp. Moreover, the availability of novel, potent compounds is predominantly confined to those that are solely effective against Gram-positive *cocci*, while their efficacy against Gram-negative bacteria remains severely constrained. Owing to ever-increasing antibiotic resistance, the limited number of available antibiotics cannot fulfill clinical challenges [12,13].

Infectious gastroenteritis is a prevalent disease worldwide, causing millions of deaths annually. In industrialized countries, it remains a major public health burden, although mortality rates are lower due to MDR gastrointestinal pathogens [14]. Pathogens such as *Clostridium difficile*, *Shigella*, *Salmonella*, *Escherichia coli*, *Vibrio cholerae*, and *Campylobacter jejuni* cause acute diarrhea [15]. Bioactive secondary metabolites represent pivotal advancements in microbial interactions and possess considerable biotechnological potential as antibiotics, biosurfactants, antivirals, and anticancer agents. The structural diversity and extensive activity spectrum of these bioactive compounds underscore the potential for novel discoveries within microbial populations [16]. Advancements in screening, separation, and isolation techniques have led to the discovery of over one million natural compounds. *Actinobacteria*, in particular, are a vital source of secondary metabolites and produce significant antimicrobial agents, such as tetracycline, vancomycin, and chloramphenicol [17]. Consequently, investigating previously unidentified microbial strains is an effective strategy for discovering novel bioactive compounds [18].

*Bacillus* species are the predominant soil bacteria owing to the formation of resistant bodies (endospores) and produce several antimicrobial substances, such as bacitracin, organic acids, diacetyl, and hydrogen, which play a significant role in inhibiting the growth of bacteria that are harmful to living organisms. Additionally, these compounds exhibit antibacterial, antibiotic, and anticancer activities [19,20]. Recent research has identified that certain metabolites, including 1,2-benzenedicarboxylic acid, exhibit anticancer properties and influence the genetic expression associated with epithelial glioblastoma [21]. This study aims to extract, identify, and characterize antimicrobial compounds produced by bacterial species in soil specimens. Furthermore, this study explored the potential application of soil bacteria in the production of secondary metabolites, with particular emphasis on their anticancer and antibacterial properties. Moreover, there is a paucity of studies examining the effects of these molecules on gene expression in cancer cells, leaving the molecular mechanisms underlying their activity largely unexplored. This constitutes a significant research gap in linking novel bioactive compounds to the specific gene-regulatory effects in cancer cells. This study further examines the impact of these molecules on gene expression in cancer cells, as well as the anticancer potential of identified compounds.

## 2. Materials and Methods

### 2.1. Isolation and Preliminary Characterization of Bacterial Samples

Following a thorough selection process of fertile farmland in Peshawar, a one-gram soil sample was collected and promptly processed in the laboratory. The soil bacteria were isolated using the serial dilution method (10^−1^ to 10^−8^) as outlined in a published report, and cultured on a nutrient agar plate at 37 °C for 24 h [22,23]. The techniques used for the initial identification of the bacterial isolates were Gram staining and enzymatic activity analysis. Biochemical tests were performed to analyze the biochemical utilization ability of the bacteria. Biochemical analyses included Indole, Urease, Triple Sugar Iron, and Citrate Utilization tests, as outlined in a published report [24]. The antimicrobial activity of the isolated bacterial strains was assessed using the spot inoculation technique, in which aliquots of bacterial cultures were spotted onto agar plates pre-inoculated with indicator microorganisms, and inhibition zones were evaluated after incubation [25].

### 2.2. PCR-16S rDNA Analysis

DNA extracted from the selected isolates was amplified via polymerase chain reaction (PCR) using 27F and 1492R primers with the sequences 16S F 5′AGAGTTTGATCCTGGCTCAG 3′ and 16S R 5′ TACGGTTACCTTGTTACGACT 3′. The reaction mixture comprised 20 μL of the master mix and 1–2 μL of the DNA sample. The PCR reaction was performed using a T100™ thermal cycler (Bio-Rad Laboratories, Inc., Hercules, CA, USA). The thermal cycling conditions included an initial denaturation at 95 °C for 5 min, followed by annealing at 55 °C for 2 min and extension at 68 °C for 1.5 min. The cycle was repeated 32 times [26].

### 2.3. Purification and Extraction of Active Metabolites

Solvent extraction was used to isolate the active metabolites from the bacterial samples. For this purpose, nutrient broth was used by inoculating the bacterial species in a 1000 mL conical flask containing nutrient broth (Thermo Scientific™ Oxoid™. CM0001, Waltham, MA, USA) and incubating it in a shaking incubator (LabTech, Daihan Labtech Co., Ltd., Namyangju, Korea) for 10 days at 37 °C. After 10 days, the flask contents were filtered to separate the bacterial biomass from the culture medium containing metabolites, as previously described [27]. The culture filtrate (200 mL) was purified three times using ethyl acetate (Sigma-Aldrich, Saint Louis, MI, USA). A 1:1 (*v*/*v*), ratio of solvent to filtrate was prepared and subjected to vigorous shaking for 3–4 days in a shaking incubator (LabTech, Daihan Labtech Co., Ltd., Namyangju, Korea). The phase containing ethyl acetate and antibiotics was separated from the aqueous phase using a separating funnel. The ethyl acetate layer was then concentrated by evaporation to dryness at 40 °C, and the resulting precipitates were further purified with methanol (Sigma-Aldrich, Saint Louis, MI, USA), yielding 0.8 g of a concentrated extract, which was brown in color [28]. The dried extract was subsequently used to assess its antibacterial activity against gastrointestinal pathogenic bacteria using the agar-well diffusion method [29].

### 2.4. Characterization of Isolated Compounds

The compounds were characterized by Gas Chromatography–Mass Spectrometry (GC-MS, Shimadzu QP2010 Ultra, Tokyo, Japan) using previously published protocols [13]. A fused silica column (30 m × 250 µm × 0.25 µm film thickness; J&W Scientific, Santa Clara, CA, USA) was utilized during the experiment. At 300 °C, three microliters of the sample were injected, and the GC run took 80 min. The helium gas (99.999% purity, Pullach, Germany) flow rate was 0.5 mL/min at a pressure of 13.7 kPa. The observed masses ranged from 28 to 600 amu, and the electron energy was 70 eV. The data obtained were compared with the National Institute of Standards and Technology (NIST) library, followed by the identification of the molecular formula, molecular weight, chemical structure, and names of the compounds [30].

### 2.5. In Vitro Biological Activities

#### 2.5.1. Antibacterial Activity

The antibacterial potential of the metabolites was assessed using the well-diffusion method, wherein 3 mg of the isolated extracts were re-dissolved in 1 mL of 1% dimethyl sulfoxide (DMSO), Sigma-Aldrich, Saint Louis, MI, USA. Using a micropipette, a total of 60 μL of the extract was introduced into each well with a 5 mm diameter. Azithromycin and DMSO were used as positive and negative controls, respectively. This procedure was conducted against gastrointestinal pathogens, including *Shigella flexneri*, *Salmonella typhi*, and *Escherichia coli*, following a previously published protocol [31] (Supplementary File Figure S3). The pathogens were isolated from clinical specimens obtained from the Mardan Medical Complex, Mardan, Pakistan.

#### 2.5.2. MTT Assay

The 3-(4, 5-dimethylthiazol-2-yl)-2, 5-diphenyltetrazolium bromide (MTT) assay was used to assess the viability and sensitivity of HCT-116 tumor cells (Sigma-Aldrich, Cat. No. CB_91091005). The cells were cultured in a 96-well plate containing RPMI 1640 medium supplemented with 10% fetal bovine serum to facilitate adhesion and proliferation. Following trypsinization to achieve a density of 1 × 10^4^ cells per well, various concentrations of ethyl acetate extracts from *B. subtilis* and *B. paralicheniformis* (ranging from 20 to 200 μg/mL) were administered. After a 48-h incubation period, MTT was added, and the cells were incubated for an additional 4 h at 37 °C. DMSO was used as a negative control. Absorbance was subsequently measured at 570 nm using a Multiskan GO spectrophotometer (Thermo Scientific, Waltham, MA, USA). Higher absorbance values correspond to increased cell viability [32]. The cells were incubated for 48 h and treated with 20, 50, 100, 150, and 200 μg/mL. Cell viability was quantified as a percentage, and the results are expressed as the mean ± standard deviation (SD).

#### 2.5.3. Assessment of Antioxidant Activity

The antioxidant activity of ethyl acetate extracts derived from *B. subtilis* and *B. paralicheniformis* was evaluated at concentrations of 20, 50, 100, 150, and 200 μg/mL using the DPPH (2,2-diphenyl-1-picryl-hydrazyl-hydrate) assay. A 0.3 mM DPPH stock solution was prepared, and 10 µL of each extract was combined with 190 µL of DPPH in a 96-well plate. Ascorbic acid was used as the positive control. Following a 30-min incubation period in the dark, the absorbance was measured at 517 nm [33]. The percentage of DPPH inhibition was calculated using the following formula:

Percentage inhibition = (absorbance of control − absorbance of sample)/absorbance of control × 100.

#### 2.5.4. Determination of Superoxide Scavenging Activity

Superoxide dismutase (SOD) activity was evaluated in HCT-116 cells treated with ethyl acetate extracts derived from *B. subtilis* and *B. paralicheniformis* at concentrations ranging from 20 to 200 μg/mL. Following cell seeding, cells were allowed to adhere before the extract was introduced into the wells. Subsequently, 20 μL of the supernatant from each experimental group was transferred to a microtiter plate, followed by the addition of 200 μL of WST solution and 20 μL of enzyme working solution. After gentle mixing, the plate was incubated at 37 °C for 20 min, and the absorbance was recorded at 450 nm. The experiments were conducted in triplicate, and the results were compared with those of the control group [34].

#### 2.5.5. Protocols for the Quantitative RT-PCR Analysis for Gene Expression

Gene expression changes in HCT-116 colorectal cancer cells were evaluated following exposure to ethyl acetate extracts of *B. subtilis* and *B. paralicheniformis* at concentrations of 100 and 200 μg/mL for 24 h using quantitative RT-PCR analysis. After treatment, the cells were centrifuged at 12,000 rpm for 5 min at 4 °C and washed three times with PBS buffer (pH 7.4) prior to total RNA extraction using TRIzol reagent (Thermo Fisher Scientific, Waltham, MA, USA). The extracted RNA was treated with RNase-free DNase I, and its concentration and purity were assessed using a NanoDrop spectrophotometer (Thermo Fisher Scientific, Waltham, MA, USA). cDNA synthesis was performed by reverse-transcribing 0.5 μg of purified RNA using the RevertAid kit (Thermo Fisher Scientific). RT-PCR amplification was conducted on a PikoReal Real-Time PCR machine (Thermo Fisher Scientific, Waltham, MA, USA), employing SYBR Green MasterMix (Applied Biosystems, Waltham, MA, USA) in a 20 μL reaction volume. Each reaction was conducted in triplicate and included a cDNA template, SYBR Green Master Mix, forward and reverse primers, and nuclease-free ddH_2_O. GAPDH mRNA levels were used for normalization [35,36].

#### 2.5.6. Statistical Analysis

Data were analyzed using SPSS software (version 20.0, IBM, Armonk, NY, USA) and expressed as mean ± standard deviation (SD). A two-way analysis of variance (ANOVA) was conducted for statistical analysis, with a significance threshold of *p* < 0.05. An independent sample t-test was employed to compare the mean antibacterial activity of the bacterial secondary metabolites with that of the standard antibiotics.

#### 2.5.7. SwissADME

The SwissADME web server (http://www.swissadme.ch/, accessed on 7 May 2026) provides an integrated platform for the rapid prediction of critical parameters, including physicochemical properties, pharmacokinetics, and drug-likeness. This tool facilitates in silico ADME/Tox profiling, a vital component of preclinical evaluation that assesses the absorption, distribution, metabolism, excretion, and toxicity of potential drug candidates to optimize their pharmacological and safety profiles.

## 3. Results

In this study, novel bacterial strains were collected, isolated, and identified from farmland in Peshawar, Pakistan. Soil bacteria were isolated using serial dilution, Gram staining, and biochemical analyses. Preliminary screening of the isolated species was performed, and based on the bioactivity results, two bacterial species (RK01 and PK04) were selected for this study. Based on Gram staining, the identified isolates were found to be Gram-positive (Supplementary File, Figure S1). After Gram staining, the samples were further identified by biochemical analysis, and the identified species, *B. paralicheniformis* and *B. subtilis*, were further confirmed by molecular identification and phylogenetic analysis (Table 1).

### 3.1. Amplification and Sequencing

The isolated strains were confirmed by PCR amplification using *16S rRNA* primers, and their size was 1200 bp (Supplementary File, Figure S2).

### 3.2. Phylogenetic Analysis Based on 16S rRNA

**Figure 1:**
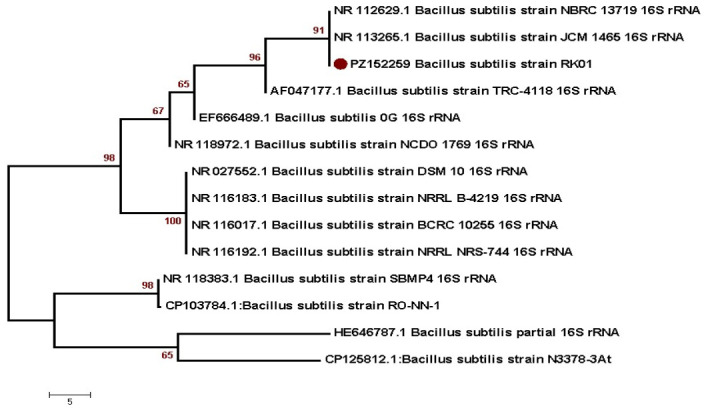
*B. subtilis* phylogenetic analysis with reference sequences by *16S rRNA* gene.

**Figure 2:**
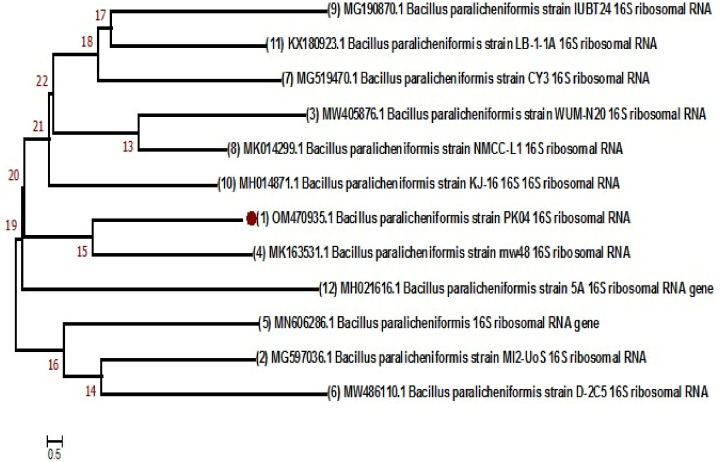
*B. paralicheniformis* phylogenetic analysis with reference sequences by *16S rRNA* gene.

Sequence data were aligned, edited, and analyzed using MEGA 7.0 before being submitted to the NCBI, which provided accession numbers for the samples (Supplementary File, Table S1). Subsequently, a phylogenetic tree was created for each isolated bacterial strain using the neighbor-joining method with similar sequences. Phylogenetic analysis indicated that the *B. subtilis* strain (RK01) with accession number PZ152259 was closely related to NR112629.1 and NR113265.1, placing them in the same clade (Figure 1). Similarly, *B. paralicheniformis* (PK04, accession number 0M470935) was closely linked to MK163531 (Figure 2). The neighbor-joining method was used to trace the evolutionary history, and the tree was scaled based on branch length. MEGA7 was used to construct a tree using the Maximum Composite Likelihood method [37].

### 3.3. Profiling of Isolated Compounds

**Figure 3:**
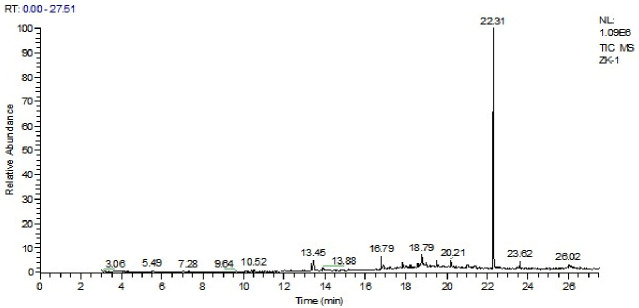
GC-MS spectrum of secondary metabolites from *B. subtilis* (RK01).

**Figure 4:**
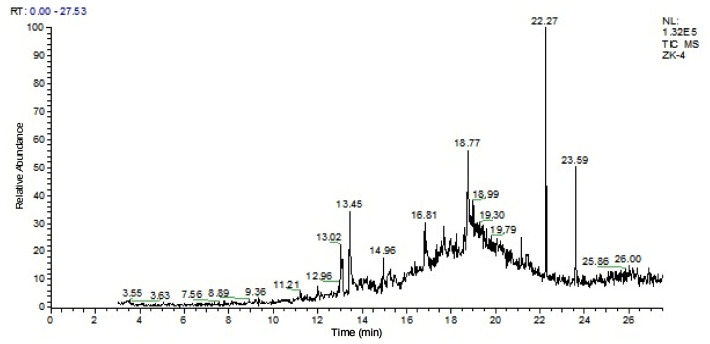
GC-MS spectrum of secondary metabolites from *B. paralicheniformis* (PK04).

Two strains, *B. subtilis* and *B. paralicheniformis*, were selected for the extraction of crude compounds using ethyl acetate [38]. GC-MS analysis elucidated the chemical composition of the *B. subtilis* extract, identifying 11 compounds (Figure 3) (Supplementary File, Table S2), while the *B. paralicheniformis* extract comprised 28 compounds (Figure 4) (Supplementary File, Table S4). Notably, many of these compounds were identified for the first time in these isolates, with some demonstrating novel identification.

### 3.4. Antibacterial Activity Against Gastrointestinal Pathogens

**Figure 5:**
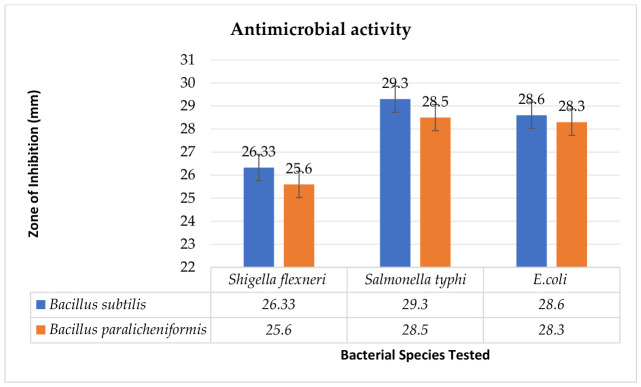
Antimicrobial activity of crude extracts of *B. subtilis* and *B. paralicheniformis*.

The antimicrobial efficacy of the crude extracts from *B. subtilis* and *B. paralicheniformis* was assessed using established protocols [39]. Following overnight incubation, the zones of inhibition (ZOI) were measured [40]. The crude extract of *B. subtilis*, at a concentration of 3 mg/mL DMSO and a volume of 60 μL, exhibited the most pronounced activity against *Shigella*, with a ZOI of 27 mm, followed by the *B. paralicheniformis* extract, which demonstrated a ZOI of 26 mm. Similarly, the *B. subtilis* extract showed significant activity against *Salmonella typhi*, with a ZOI of 30 mm, whereas the *B. paralicheniformis* extract exhibited a ZOI of 29 mm. In contrast to *E. coli*, the *B. subtilis* extract produced a 29 mm ZOI, while the *B. paralicheniformis* extract produced a ZOI of 28 mm (Table 2 and Figure 5).

### 3.5. MTT Assay

**Figure 6:**
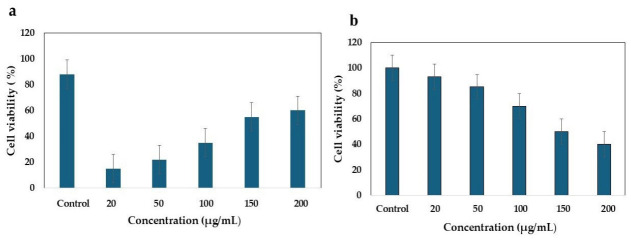
(**a**) Cytotoxic effect of various concentrations of EA-Bs on the proliferation of HCT-116 colorectal cancer cells. (**b**) The cytotoxic impact of various concentrations of EA-Bp on the proliferation of HCT-116 colorectal cancer cells. The results illustrate a reduction in percent cell viability with increasing concentration. The experiment was conducted in triplicate, and the mean values of the triplicate measurements were utilized.

The present study evaluated the cytotoxic effects of ethyl acetate extracts derived from *B. subtilis* and *B. paralicheniformis* on HCT-116 colorectal carcinoma cells. The mitochondrial function of the cells was assessed using an MTT assay protocol [32]. All tested extracts exhibited significant cytotoxic activity compared to the control, indicating that *B. subtilis* and *B. paralicheniformis* demonstrated dose-dependent inhibition of tumor cell growth across a concentration range of 20–200 μg/mL. Notably, the *B. subtilis* extract exhibited a higher cytotoxic response than the extracts of other bacterial species under investigation at all concentrations (Figure 6). Furthermore, both *B. paralicheniformis* and *B. subtilis* extracts followed a similar dose-dependent trend, albeit with reduced potency. The cytotoxic effects of varying concentrations of the ethyl acetate extracts of *B. subtilis* (EA-Bs) on the proliferation of HCT-116 colorectal cancer cells are shown in Figure 6a. It is noteworthy that an increase in extract concentration corresponded with a reduction in the viability of HCT-116 cells (EA-Bs20 µg/mL: 87.55 ± 3.54, EA-Bs-50 µg/mL: 77.87 ± 3.1, EA-Bs-100 µg/mL: 52.47 ± 2.95, EA-Bs-150 µg/mL: 41.28 ± 1.9, EA-Bs-200 µg/mL: 29.4 ± 1.42). The EA-Bs extract demonstrated significant cytotoxicity, reducing colorectal cancer cell viability to 29.4% at a concentration of 200 μg/mL, as illustrated in Figure 6b. Conversely, the lowest concentration (20 μg/mL) exhibited minimal cytotoxic activity (87.55 ± 3.54). In comparison, the cytotoxic effects of different concentrations of *B. paralicheniformis* (EA-Bp) on HCT-116 colorectal cancer cells revealed that at the lowest concentration (EA-Bs-20 µg/mL), cell viability was 90.8 ± 6.82%. As the concentration increased to 50 µg/mL, cell viability further decreased to 85.27 ± 2.26%. This trend persisted with increasing concentrations (EA-Bp-20 µg/mL: 90.8 ± 6.82, EA-Bp-50 µg/mL: 85.27 ± 2.26). Both samples exhibited dose-dependent inhibitory effects, and the IC_50_ values for EA-Bs and EA-Bp were determined to be 150 µg/mL, corresponding to the concentration at which 50% inhibition of activity was observed.

### 3.6. DPPH Assay for Antioxidant Activity

**Figure 7:**
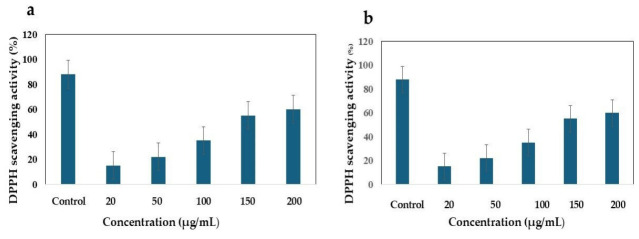
(**a**) DPPH radical scavenging activity of EA-Bs. (**b**) DPPH radical scavenging activity of EA-Bp. The concentrations of the EA-Bs and EA-Bp extracts were 20, 50, 100, 150, and 200 μg/mL, respectively. Ascorbic acid was used as a positive control. The incubation period was 30 min, and activity was measured as a percentage. The experiment was conducted in triplicate, and the mean values of the triplicate measurements were utilized.

The DPPH assay was used to assess the free-radical-scavenging potential and antioxidant properties. In this study, the DPPH assay was used to measure the free radical scavenging capacity of EA-Bs and EA-Bp. The sample concentrations for the DPPH assay ranged from 20 to 200 μg/mL. Compared to the control samples, the tested samples exhibited notable radical-scavenging activity. Among the concentrations tested, the EA-Bs extract demonstrated superior DPPH scavenging activity compared to other bacterial samples. Both EA-Bp and EA-Bs samples followed this trend, albeit with reduced potency. The radical scavenging activity of the extracts was expressed as a percentage (%), and the data are presented as the mean ± SD. The DPPH scavenging activity of the EA-Bs extract at various concentrations is detailed as follows: EA-Bs: (Positive control-ascorbic acid): 78.15 ± 4.02, EA-Bs-20 µg/mL: 11.35 ± 0.58, EA-Bs-50 µg/mL: 25.83 ± 2.13, EA-Bs-100 µg/mL: 47.05 ± 2.1, EA-Bs-150 µg/mL: 58.91 ± 3.71, EA-Bs-200 µg/mL: 74.13 ± 2.7. At a concentration of 20 μg/mL, EA-Bs exhibited a scavenging activity of 11.35 ± 0.58%. As the concentration increased to 50 μg/mL, the activity improved to 25.83 ± 2.13%. Further increases in concentration (100, 150, and 200 μg/mL) resulted in enhanced scavenging activity, reaching 47.05 ± 2.1%, 58.91 ± 3.71%, and 74.13 ± 2.7%, respectively (Figure 7a). The DPPH scavenging activity of the EA-Bp extract at various concentrations was as follows: EA-Bp (positive control-ascorbic acid): 78.15 ± 4.02, EA-Bp-20 µg/mL: 9.44 ± 0.89, EA-Bp-50 µg/mL: 25.98 ± 2.03, EA-Bp-100 µg/mL: 41.5 ± 3.44, EA-Bp-150 µg/mL: 50.42 ± 4.12, EA-Bp-200 µg/mL: 66.12 ± 3.12. Similarly, EA-Bp demonstrated concentration-dependent DPPH radical scavenging activity. At a concentration of 20 μg/mL, the scavenging activity was recorded at 9.44 ± 0.89%. As the concentration increased to 50 μg/mL, the activity rose to 25.98 ± 2.03%. Further increases in concentration (100, 150, and 200 μg/mL) resulted in enhanced scavenging activity, reaching 41.5 ± 3.44%, 50.42 ± 4.12%, and 66.12 ± 3.12%, respectively (Figure 7b).

### 3.7. Superoxide Scavenging Activity

**Figure 8:**
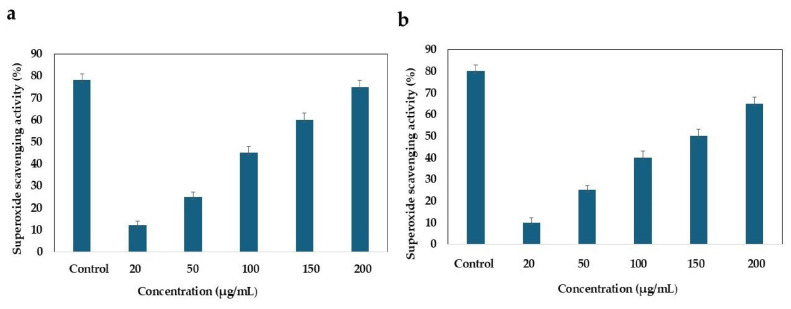
(**a**) Evaluation of SOD activity of HCT-116 colorectal cancer cells treated with EA-Bs. (**b**) Evaluation of SOD activity of HCT-116 colorectal cancer cells treated with EA-Bp. Ascorbic acid was used as a positive control. The SOD activity was measured as a percentage. The data is reported as mean ± SD. The experiment was conducted in triplicate, and the mean values of the triplicate measurements were utilized.

SOD is an essential antioxidative enzyme within cells that is responsible for converting superoxide ions into less harmful products. Notably, cancerous cells exhibited reduced SOD activity. In this study, we observed an increase in SOD activity in HCT-116 cells treated with varying concentrations of EA-Bs and EA-Bp. The concentrations tested were 20, 50, 100, 150, and 200 μg/mL. The antioxidant SOD activity of each extract at these concentrations is described as follows: For EA-Bs: (positive control: 87.19 ± 5.35, EA-Bs-20 µg/mL: 13.84 ± 0.85, EA-Bs-50 µg/mL: 23.14 ± 1.35, EA-Bs-100 µg/mL: 36.76 ± 1.21, EA-Bs-150 µg/mL: 56.62 ± 2.82, EA-Bs-200 µg/mL: 62.05 ± 2.62). The EA-Bs demonstrated significant SOD activity across the tested concentrations (Figure 8), with an increase in activity in a concentration-dependent manner, suggesting the presence of bioactive compounds capable of scavenging superoxide radicals. EA-Bs exhibited SOD activity of 62.05 ± 2.62% at the highest concentration (200 µg/mL). For EA-Bp: (positive control: 87.19 ± 5.35, EA-Bp-20 µg/mL: 10.93 ± 1.16, EA-Bp-50 µg/mL: 19.29 ± 1.56, EA-Bp-100 µg/mL: 38.21 ± 2.55, EA-Bp-150 µg/mL: 43.13 ± 3.23, EA-Bp-200 µg/mL: 54.04 ± 3.04. It was observed that EA-Bp exhibited SOD activity that varied with concentration (Figure 8a,b), with activity levels increasing as the concentration increased. At the highest concentration (200 µg/mL), EA-Bp demonstrated an SOD activity of 54.04 ± 3.04%. These findings suggested that EA-Bp contains bioactive compounds with antioxidant properties.

### 3.8. Quantitative RT-PCR Analysis for Gene Expression

**Figure 9:**
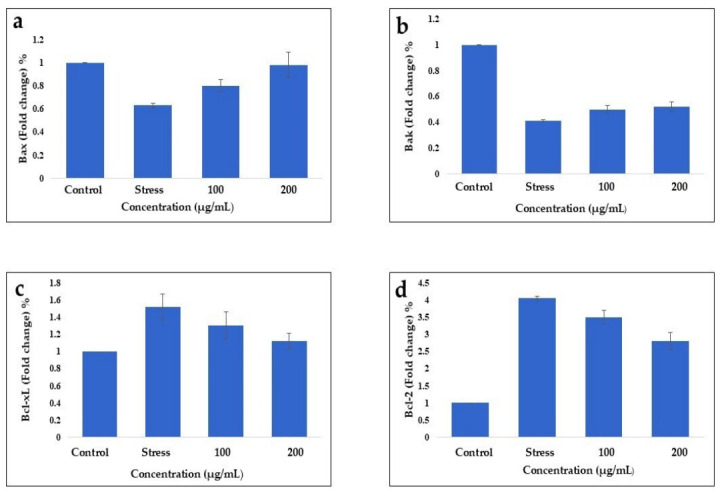
The mRNA quantitative RT-PCR expression studies of apoptotic (*Bax* and *Bak*) and anti-apoptotic (*Bcl-2* and *Bcl-x_L_*) genes in HCT-116 colorectal cancer cells treated with EA-Bs extract at 100 µg/mL and 200 µg/mL concentrations. GAPDH was used as an internal control. The stress was induced in HCT-116 cells using Cobalt chloride (CoCl_2_). Data was normalized with internal control. The data is reported as mean ± SD. The experiment was conducted in triplicate, and the mean values of the triplicate measurements were utilized. (**a**) Indicates fold change expression of *Bax*: (Stress: 0.626 ± 0.017, EA-Bs-100 µg/mL: 0.973 ± 0.049, EA-Bs-200 µg/mL: 1.251 ± 0.107). (**b**) shows mRNA levels of *Bak*: (Stress: 0.408 ± 0.025, EA-Bs-100 µg/mL: 0.551 ± 0.027, EA-Bs-200 µg/mL: 0.735 ± 0.034). (**c**) Represent fold change expression of *Bcl-xL*: (Stress: 1.638 ± 0.153, EA-Bs-100 µg/mL: 1.415 ± 0.162, EA-Bs-200 µg/mL: 1.224 ± 0.095). (**d**) shows mRNA expression levels of *Bcl-2*: (Stress: 4.112 ±0.087, EA-Bs-100 µg/mL: 3.714 ± 0.194, EA-Bs-200 µg/mL: 2.274 ± 0.203.

While investigating the radical scavenging, cytotoxic, and SOD activities of the bacterial extracts, EA-Bs and EA-Bp demonstrated promising results. The mRNA expression levels of genes involved in cell cycle regulation in colorectal cancer cells, specifically HCT-116, were analyzed using quantitative reverse transcription polymerase chain reaction (RT-PCR). The cells were subjected to a stress induction test with cobalt chloride (CoCl_2_), followed by treatment with EA-Bs extracts at concentrations of 100 µg/mL and 200 µg/mL, using GAPDH as an internal control. The data were normalized accordingly. *Bax* and *Bak* were identified as apoptotic genes, while *Bcl-2* and *Bcl-xL* were identified as anti-apoptotic genes. The mRNA expression levels of *Bax* and *Bak* were assessed in response to the different treatments. Figure 9 highlights variations in gene expression in HCT-116 cells treated with EA-Bs. The apoptotic genes *Bak* and *Bax* were significantly elevated in HCT-116 cells compared with those in the stress group. EA-Bs significantly upregulated the expression of these genes in a dose-dependent manner (Figure 9a,b). The expression of *Bax* was significantly lower in the stress group than in the control group (stress: 0.626 ± 0.017). However, treatment with EA-Bs at both 100 µg/mL (0.973 ± 0.049) and 200 µg/mL (1.251 ± 0.107) concentrations resulted in the upregulation of *Bax* expression (Figure 9a). A similar trend was observed for *Bak*. The stress condition resulted in basal levels of *Bak* expression (0.408 ± 0.025). Treatment with EA-Bs100 µg/mL (0.551 ± 0.027) and 200 µg/mL (0.735 ± 0.034) significantly increased *Bak* expression (Figure 9b). Furthermore, the mRNA levels of anti-apoptotic genes (*Bcl-2* and *Bcl-xL*) were examined at 100 and 200 μg/mL. These concentrations significantly downregulated anti-apoptotic genes compared to the stress group. In the case of *Bcl-2*, the mRNA expression was downregulated to 3.714 ± 0.194 and 2.274 ± 0.203, respectively (Figure 9d). The EA-Bp extract demonstrated a suppressive effect on *Bcl-xL* expression at 100 µg/mL (1.415 ± 0.162) and 200 µg/mL (EA-Bs: 1.224 ± 0.095) (Figure 9c).

**Figure 10:**
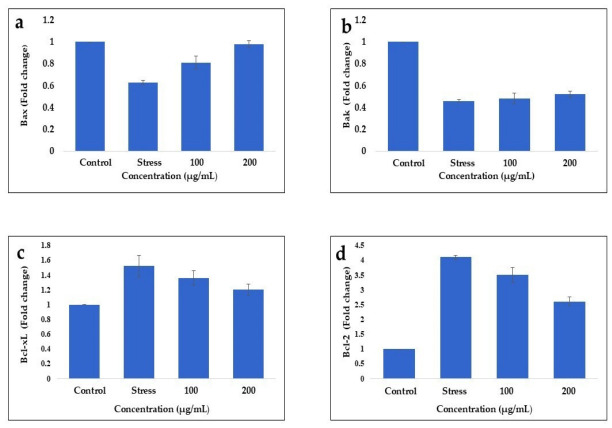
The mRNA quantitative RT-PCR expression studies of apoptotic (*Bax* and *Bak*) and anti-apoptotic (*Bcl-2* and *Bcl-x_L_*) genes in HCT-116 colorectal cancer cells treated with EA-Bp at 100 and 200 µg/mL. GAPDH was used as an internal control. The stress was induced in HCT-116 cells using Cobalt chloride (CoCl_2_). Data was normalized with internal control. The data is reported as mean ± SD. The experiment was conducted in triplicate, and the mean values of the triplicate measurements were utilized. (**a**) indicates fold change expression of *Bax*: (Stress: 0.626 ± 0.017, EA-Bp-100 µg/mL: 0.805 ± 0.056, EA-Bp-200 µg/mL: 0.982 ± 0.031). (**b**) shows mRNA levels of *Bak*: (Stress: 0.408 ± 0.025, EA-Bp-100 µg/mL: 0.487 ± 0.048, EA-Bp-200 µg/mL: 0.566 ± 0.022). (**c**) represent fold change expression of *Bcl-xL*: (Stress: 1.638 ± 0.153, EA-Bp-100 µg/mL: 1.368 ± 0.103, EA-Bp-200 µg/mL: 1.036 ± 0.087). (**d**) shows mRNA expression levels of *Bcl-2*: (Stress: 4.112 ± 0.087, EA-Bp-100 µg/mL: 3.545 ± 0.297, EA-Bp-200 µg/mL: 2.611 ± 0.152).

Treatment with EA-Bp resulted in the upregulation of *Bax* expression at 100 µg/mL (0.805 ± 0.056) and 200 µg/mL (0.982 ± 0.031) (Figure 10a). Similarly, treatment with 100 µg/mL (0.487 ± 0.048) and 200 µg/mL (0.566 ± 0.022) of EA-Bp led to an increase in *Bak* expression compared with the stress condition (Figure 10b). The expression levels of the anti-apoptotic genes *Bcl-2* and *Bcl-xL* were also evaluated. Under stress conditions, there was a notable elevation in *Bcl-2* (4.112 ± 0.087) and *Bcl-xL* (1.638 ± 0.153) expression levels compared to those in the control. Treatment with 100 µg/mL (3.545 ± 0.297) and 200 µg/mL (2.611 ± 0.152) of EA-Bp resulted in a reduction in *Bcl-2* expression (Figure 10d). Furthermore, the EA-Bp extract demonstrated a suppressive effect on *Bcl-xL* expression at 100 µg/mL (1.368 ± 0.103) and 200 µg/mL (1.036 ± 0.087) compared to the stress group (Figure 10c).

### 3.9. Pharmacokinetic Drug Likeness Properties

We also attempted to calculate some of the important properties of the compounds using well-known computational software (SwissADME), and the results are listed (Supplementary File, Tables S3 and S5. The pharmacokinetic characteristics showed that all the targets were predicted to possess good GI absorption; therefore, they did not cause any problems in the excretion of the drug, and most of the compounds exhibited BBB permeation. Their negative log Kp values ranged from −7.93 to −3.02, indicating the lowest ability to penetrate the skin. The drug-likeness parameters of the title compounds can be easily assessed from bioavailability radar plots by using the optimal range for each property (LIPO, size, polarity, solubility, and flexibility).

## 4. Discussion

The increasing prevalence of antibiotic-resistant bacteria necessitates the discovery and development of novel antimicrobial agents. Bacterial secondary metabolites have attracted attention because of their specific antimicrobial properties and complex chemical structures. The genus *Bacillus*, predominantly found in soil, is recognized as a significant source of bioactive natural compounds and offers a diverse array of valuable natural products [41]. In this study, two bacterial strains, *B. subtilis* and *B. paralicheniformis*, were identified using biochemical and molecular techniques. Soil samples were subjected to serial dilution for isolation, and bacteria were cultured on nutrient agar at 37 °C overnight. Identification was accomplished using Gram staining and enzymatic activity tests. DNA was extracted from fresh colonies, and *16S rRNA* primers were used for PCR amplification to confirm the identification. The PCR products were analyzed for phylogenetic properties following a methodology similar to that of Gowsalya et al. (2014), with the addition of phylogenetic analysis [42]. The diverse structures and biological significance of microbial metabolites, including their anti-tumor and antimicrobial activities, underscore their importance in research [5]. This study emphasizes the extraction and characterization of secondary metabolites from two bacterial species using GC-MS analysis. Ethyl acetate was used as the extraction solvent, and a previously reported method was used with slight modifications [43]. Ghadin et al. (2008) suggested that ethyl acetate is more effective than other solvents against bacterial pathogens, a finding corroborated by our laboratory results when used at a 1:1 ratio [44].

In this study, we identified 11 compounds from *B. subtilis* and 28 compounds from *B. paralicheniformis*. Identification was based on parameters such as retention time, molecular weight, peak area percentage, and molecular formula using the *NIST* library. The characterized compounds were benzene, nitro-, acetamide, *N*-methyl-*N*-[4-[2 acetoxymethyl-1-pyrr olidyl]-2-butynyl]-, tetradecane, hexadecane, acetamide, *N*-(4-phenylbutyl)-, 2(3H)-furanone, dihydro-5-tetradecyl, 17-pentatriacontene, dodecanecarboxamide, *N*-[2-(3-indolyl)ethyl]-, erucic acid, 2,2,4-trimethyl-3-(3,8,12,16-tetrameth yl-heptadeca-3,7,11,15-tetraenyl)-cycloh exanol, tert hexadecanethiol, 9-heptadecanone, 1-chloroeicosane, corynan-17-ol,18,19-didehydro-10- methoxy-, and acetate (ester). These compounds have been reported in the previous literature to possess antibacterial and anticancer properties. A similar study conducted by Jalaluldeen et al. (2015) successfully identified 77 compounds from *Actinomycetes* using GC-MS analysis [45]. Nas et al. (2021) [46] employed the same extraction and characterization techniques to identify 56 compounds, focusing on halotolerant *Bacillus* spp. from the Great Sebkha of Oran. The characterized compounds included *tert*-butyl phenol compounds, fatty acid methyl esters owing to the methylation procedure, hydrocarbons, aldehydes, benzoquinones, pyrroles, and terpenes. Previous studies have reported that such compounds have wide biological and pharmaceutical applications [46]. Most of the identified compounds exhibited antibacterial activity against gastrointestinal pathogens such as *Salmonella*, *Shigella*, and *E. coli*, demonstrating significant inhibition zones in the well diffusion method. In contrast, Khattab et al. (2016) isolated compounds from two different *Streptomyces* strains using GC-MS and assessed their antibacterial effects against *Staphylococcus aureus* and *Escherichia coli* [47]. Another focus of our study was to evaluate the anticancer activities of the isolated compounds. Cancer is a major global health concern and a leading cause of death among noncommunicable diseases, and many synthetic drug molecules have been approved by the U.S. Food and Drug Administration (FDA) [48,49,50,51]. In addition to synthetic compounds, natural compounds from plants, fungi, bacteria, and marine organisms are vital sources of anti-cancer agents. This study underscores the necessity of screening extracts from various microbial sources to identify potential anticancer drug candidates. These findings indicate that the extracts of *B. subtilis* and *B. paralicheniformis* can inhibit the growth of colorectal tumor cells. These microbes are promising sources of bioactive compounds with anticancer properties, warranting further research to identify compounds responsible for these effects. Such investigations could aid the development of new cancer therapies. This study has limitations, including the use of a single cell line and a lack of in vivo analysis. These current research outcomes align with previous research that outlined the anticancer effects of microbial metabolites, including lipopeptides and non-ribosomal peptides, which are active against colorectal cancer. The DPPH assay was employed to evaluate the antioxidant potential of the extract samples through their free radical scavenging ability. This study demonstrated the antioxidant potential (scavenging ability) of *B. subtilis* and *B. paralicheniformis* at all concentrations, with the ethyl acetate extract containing the antioxidant component. The scavenging potential is likely attributable to the presence of phenolic compounds, flavonoids, or other bioactive compounds in the extracts [52]. This study highlighted varying levels of SOD activity, with *B. subtilis* exhibiting the highest SOD activity level. The dose-dependent increase in SOD activity in both *B. subtilis* and *B. paralicheniformis* indicates the presence of bioactive antioxidant compounds. Future studies should aim to identify and characterize these compounds for therapeutic applications and investigate the underlying mechanisms. Some bacterial metabolites have been shown to exhibit SOD and DPPH activities in colorectal cancer cells and in animal models. Zhang et al. (2019) reported that a polysaccharide from *Bifidobacterium longum* enhanced SOD activity in a rat model of colorectal cancer and inhibited the growth and invasion of HCT-116 colorectal cells in vitro [39].

Similarly, Wang et al. (2024) [53] evaluated superoxide dismutase 3 (SOD3) expression and its clinical relevance in colorectal cancer using nude mice. We found that SOD3 overexpression significantly reduced Ki67 expression (*p* < 0.01), inhibited tumor development (*p* < 0.01), and prevented liver metastasis (*p* < 0.001). This study focused on the mRNA expression of apoptotic and anti-apoptotic genes in HCT-116 cells treated with *B. subtilis* and *B. paralicheniformis*. Our study showed that both extracts influenced the expression of these genes in colorectal cancer cells. The response of Bax and Bak to the extracts suggested their potential enhancement in HCT-116 cells. Bax and Bak promote mitochondrial outer membrane permeabilization and the release of cytochrome c, leading to apoptosis. This indicates that *B. subtilis* and *B. paralicheniformis* extracts could activate the mitochondrial apoptotic pathway. The downregulation of anti-apoptotic genes (*Bcl-2* and *Bcl-xL*) supports this hypothesis because these genes inhibit cytochrome release and maintain mitochondrial integrity. Overexpression of *Bcl-2* and *Bcl-xL* is often associated with increased cell survival and resistance to apoptosis in cancer. Many microbial compounds, such as iturin, fengycin, surfactin, and bacillomycin D, have been shown to induce positive responses to apoptosis in colorectal cancer cells. Among the microbial compounds, Surfactin enhances apoptosis in LoVo cells by upregulating FasR, FasL, Cleaved PARP, and p21waf1/cip1, while downregulating CDK2 and cyclin E2. It has been shown that iturin A induces apoptosis in Caco-2 cells by increasing Bax and Bad expression and downregulating *Bcl-2* expression, leading to cytochrome c release and elevated ROS levels. Moreover, fengycin triggered apoptosis in HT29 cells by upregulating caspase-3, caspase-6, and Bax, and downregulating Bcl-2 and CDK4/cyclin D1. Our findings support the potential use of *B. subtilis* and *B. paralicheniformis* extracts as therapeutic agents against colorectal cancer. However, further studies are required to elucidate the underlying molecular mechanisms. Moreover, the study has limitations due to the lack of a standard chemotherapeutic agent serving as a positive control in both the MTT and gene expression assays. Additionally, the study did not determine the minimum inhibitory concentration (MIC) and minimum bactericidal concentration (MBC) for assessing antibacterial activity. These issues are planned to be addressed in subsequent research efforts.

## 5. Conclusions

The present study demonstrated the significance of soil as a preferred habitat and examined the influence of culture conditions on the production of secondary metabolites. This study further demonstrated that these compounds exhibited substantial antibacterial activity when tested against gastrointestinal pathogens, including *Salmonella*, *Shigella*, and *Escherichia coli*. Additionally, we elucidated the value of these natural products, emphasizing the importance of bacterial metabolites in cancer therapy. Based on *16S rRNA* gene sequencing and biochemical tests, bacterial strains identified as *B. subtilis* and *B. paralicheniformis* were analyzed. These extracts demonstrated high inhibitory activity against gastrointestinal pathogens, such as *Salmonella* Typhi, *Shigella flexneri*, and *E. coli* through a well diffusion method. Additionally, the MTT assay indicated the anticancer effects of the extracts by suppressing the growth of colorectal tumor cells, warranting further exploration using GC-MS. The DPPH assay revealed that *B. subtilis* and *B. paralicheniformis* exhibited the highest free radical scavenging activity at all concentrations tested. *B. subtilis* also showed the highest SOD activity, indicating the presence of bioactive antioxidant compounds. Additionally, this study noted changes in the expression of apoptotic and antiapoptotic genes in HCT-116 cells treated with these extracts. Upregulation of Bax and Bak was observed, suggesting a correlation with apoptotic response. The current study is limited to in vitro investigations; future research should prioritize in vivo testing with suitable animal models to confirm the safety, bioavailability, and therapeutic efficacy of bioactive compounds.

## Figures and Tables

**Table 1:** Biochemical analysis of the two isolates, *Bacillus subtilis* (*B. subtilis*) and *Bacillus paralicheniformis* (*B. paralicheniformis*).

Sr. No	Description	Gram Staining	Catalase	Oxidase	Indole	Citrate	Urease	Identified
1	EA-Bs	+	+	+	-	+	-	*B. subtilis*
2	EA-Bp	+	+	-	-	-	+	*B. paralicheniformis*

+ = *Positive*, - = *Negative*, Ethyl acetate extracts of *B. subtilis* = EA-Bs, Ethyl acetate extracts of *B. paralicheniformis =* EA-Bp.

**Table 2:** Antimicrobial activity of crude extracts of *B. subtilis* and *B. paralicheniformis*.

Sr. No	Bacterial Extract	ZOI of Extract (mm)-Mean		ZOI of Azithromycin (mm)	DMSO
		*B. subtilis*	*B. paralicheniformis*		
1	*Shigella flexneri*	26.3 ± 0.57	25.6 ± 0.57	14	0
2	*Salmonella typhi*	29.3 ± 0.57	27.6 ± 0.57	17.9	0
3	*Escherichia coli*	28.6 ± 0.57	28.3 ± 0.57	15.8	0

Data are represented as the mean ± SD (*n* = 3), significantly different at *p* < 0.05.

## Data Availability

The original contributions of this study are included in the article/Supplementary Material. Further inquiries should be directed to the corresponding authors.

## Supplementary Materials

The following supporting information can be downloaded at: https://www.mdpi.com/article/10.3390/cimb48050514/s1.
