# Supplementary material for: Molecular Profiling of Soil-Derived Bacillus subtilis and Bacillus paralicheniformis: Evaluation of Anticancer and Antibacterial Efficacy Against Gastrointestinal Pathogens
Source: Curr Issues Mol Biol. 2026 May 15;48(5):514. doi: 10.3390/cimb48050514 (PMC13206219; doi:10.3390/cimb48050514)
Supplement: Supplementary file 1 [file cimb-48-00514-s001.zip › cimb-4277218-supplementary.pdf]

Molecular Profiling of Soil-Derived *Bacillus subtilis* and *Bacillus paralicheniformis*: Evaluation of Anticancer and Antibacterial Efficacy against Gastrointestinal Pathogens

Rida Kiyani [1], Komal Aman [2], Tajamul Hussain [3], Hazir Rahman [1], Ziaur Rahman [1,*], Ahmed Muhammad Ajaz [4], Muhammad Latif [5,6,7,*], Salman Alrokayan [8]

[1] Department of Microbiology, Abdul Wali Khan University, Mardan- 23200, Khyber Pakhtunkhwa, Pakistan; ridakayani04@gmail.com (R.K.), hazirrahman@awkum.edu.pk (H.R.), zrahman@awkum.edu.pk (Z.R.)
[2] Department of Microbiology, Women University Mardan, Khyber Pakhtunkhwa, Pakistan; Dr.komalaman@wumardan.edu.pk (K.A.)
[3] Center of Excellence in Biotechnology Research, King Saud University, Riyadh 11451, Saudi Arabia; thussain@ksu.edu.sa (T.H.)
[4] Institute of Green-Bio Science and Technology, Seoul National University, PyeongChang 232-916, Republic of South Korea; Ajaz; m.ajaz@snu.ac.kr (A.M.A)
[5] Department of Chemistry, Rawalpindi Women University (RWU), Rawalpindi, Punjab 43600, Pakistan; latifmayo@gmail.com (M.L.)
[6] Centre for Genetics and Inherited Diseases (CGID), Taibah University, Madinah, Saudi Arabia; latifmayo@gmail.com (M.L.)
[7] Department of Basic Medical Sciences, College of Medicine, Taibah University, Madinah, Saudi Arabia; latifmayo@gmail.com (M.L.)
[8] Biochemistry Department, College of Science, King Saud University, Riyadh 11451, Saudi Arabia; salrokayan@ksu.edu.sa (S.A.)
* Correspondence: Zrahman@awkum.edu.pk (Z.R.), latifmayo@gmail.com (M.L.)

Table S1. Sequence analysis of soil isolates.

Sr. No	Title	Accession Number	URLS
1	*Bacillus subtilis* strain RK01	PZ152259	https://www.ncbi.nlm.nih.gov/nuccore/PZ152259.1/
2	*B. paralicheniformis* strain PK04	OM470935	https://www.ncbi.nlm.nih.gov/nuccore/OM470935

Table S2. Newly identified compounds of *B. subtilis* (RK01)

S. No	Compound name	RT (Min)	Area %	Molecular formula	Molecular weight (g/mol)	Chemical origin
1	Acetamide, *N*-methyl-*N*-[4-[2-acetoxymethyl-1-pyrr olidyl]-2-butynyl]-	9.62	0.15	$C_{14}H_{22}N_2O_3$	266	Atenolol/beta-blocker
2	Acetamide, *N*-(4-phenylbutyl)-	13.92	3.16	$C_{12}H_{17}NO$	191	Benzamide
3	Ethanol, 2-(tetradecyloxy)-	15.20	0.77	$C_{16}H_{34}O_2$	258	Cetyl glycol
4	2(3*H*)-Furanone, dihydro-5-tetradecyl	15.92	0.15	$C_{18}H_{34}O_2$	282	Eliadate/ oleic Acid
5	2-Nonadecanone 2,4-dinitrophenylhydrazine Not reported	17.20	0.77	$C_{25}H_{42}N_4O_4$	462	Hydrazone
6	3-Pyridinecarboxylic acid, 2,7,10-tris(acetyloxy)-1,1a,2,3,4,6,7,10,11,11a-decahydro-1,1,3,6,9-pentamethy l-4-oxo-4a,7a-epoxy-5H-cyclopenta[a]cy clopropa[f]cycloundecen-11-yl ester,[1aR(1aR*,2R*,3S*,4aR*,6S*,7S*,7aS *,8E,10R*,11R*,11aS*)]-	20.56	0.11	$C_{32}H_{39}NO_{10}$	597	Complex organic compound
7	Dodecanecarboxamide, *N*-[2-(3-indolyl) ethyl]-	21.05	1.77	$C_{22}H_{34}N_2O$	342	Indole amide
8	18,19-Secoyohimban-19-oic acid, 16,17,20,21-tetradehydro-16-(hydroxy methyl)-, methyl ester, (15á,16E)-	21.99	0.14	$C_{21}H_{24}N_2O_3$	352	Indole alkaloid

Table S2. Newly identified compounds of *B. subtilis* (RK01) (***Continued***)

S. No	Compound name	RT (Min)	Area %	Molecular formula	Molecular weight (g/mol)	Chemical origin
9	2,2,4-Trimethyl-3-(3,8,12,16-tetrameth yl-heptadeca-3,7,11,15-tetraenyl)-cycloh exanol	23.62	2.08	$C_{30}H_{52}O$	428	Terpenes alcohols
10	Bacteriochlorophyll-c-stearyl	16.81	3.39	$C_{52}H_{72}MgN_4O_4$	840	Porphyrin
11	Butanoic acid, 1a,2,5,5a,6,9,10,10a-octahydro-5,5a-di hydroxy-4-(hydroxymethyl)-1,1,7,9-tetra methyl-11-oxo-1H-2,8a-methanocyclop enta[a]cyclopropa[e]cyclodecen-6-yl ester, [1aR-(1aà,2à,5á,5aá,6á,8aà,9à,10aà)]-	27.28	0.13	$C_{24}H_{34}O_6$	418	Natural product ester

Table S3. Pharmacokinetic properties of *B. subtilis* (RK01) isolate.

S. No	Compound name	Flexibility	TPSA (Polarity) (Å²)	Bioavailability (F)	XlogP3 (Lipophilicity)	GI	BBB	Solubility	LogK_p (cm/s)
1	Acetamide, *N*-methyl-*N*-[4-[2-acetoxymethyl-1-pyrr olidyl]-2-butynyl]-	3	23.55	0.55	0.31	High	No	Very soluble	-7.26
2	Acetamide, *N*-(4-phenylbutyl)-	5	29.10	0.55	3.30	High	Yes	Soluble	-5.12
3	Ethanol, 2-(tetradecyloxy)-	15	29.46	0.55	6.19	High	Yes	Moderately soluble	-3.48
4	2(3*H*)-Furanone, dihydro-5-tetradecyl	13	26.30	0.55	7.05	High	Yes	Moderately soluble	-3.02
5	2-Nonadecanone 2,4-dinitrophenylhydrazine<br>Not reported	20	116.03	0.55	11.46	Low	No	Poorly soluble	-0.99
6	3-Pyridinecarboxylic acid, 2,7,10-tris(acetyloxy)-1,1a,2,3,4,6,7,10,11,11a-decahydro-1,1,3,6,9-pentamethy l-4-oxo-4a,7a-epoxy-5H-cyclopenta[a]cy clopropa[f]cycloundecen-11-yl ester,[1aR(1aR*,2R*,3S*,4aR*,6S*,7S*,7aS *,8E,10R*,11R*,11aS*)]-	9	147.69	0.17	2.84	Low	No	Moderately soluble	-7.93

Table S3. Pharmacokinetic properties of *B. subtilis* (RK01) isolate *(**Continued**).*

S. No	Compound name	Flexibility	TPSA (Polarity) (Å²)	Bioavailability (F)	XlogP3 (Lipophilicity)	GI	BBB	Solubility	LogK_p (cm/s)
7	Dodecanecarboxamide, N-[2-(3-indolyl)ethyl]-	14	44.89	0.55	6.12	High	Yes	Moderately soluble	-4.04
8	18,19-Secoyohimban-19-oic acid, 16,17,20,21-tetradehydro-16-(hydroxy methyl)-, methyl ester,	4	45.33	0.55	4.11			Moderately soluble	-5.46
9	2,2,4-Trimethyl-3-(3,8,12,16-tetramethyl-heptadeca-3,7,11,15-tetraenyl)-cycloh exanol	12	20.23	0.55	10.41			Poorly soluble	-1.52
10	Bacteriochlorophyll-c-stearyl	23	89.38	0.56	10.84			Insoluble	-3.74
11	Butanoic acid, 1a,2,5,5a,6,9,10,10a-octahydro-5,5a-di hydroxy-4-(hydroxymethyl)-1,1,7,9-tetra methyl-11-oxo-1H-2,8a-methanocyclop enta[a]cyclopropa[e]cyclodecen-6-yl ester, [1aR-(1aà,2à,5á,5aá,6á,8aà,9à,10aà)]-	5	100.90	0.55	1.86	High	No	Soluble	-7.52

Table S4. Newly identified compounds of *B. paralicheniformis* (PK04).

S. No	Compound name	RT (Min)	Area %	Molecular formula	Molecular weight (g/mol)	Chemical origin
1	Phenanthro[1,2-c][1,2,5]selenadiazoe	3.49	0.67	$C_{14}H_8N_2Se$	484	Heterocyclic aromatic compound
2	Decanoic acid, octadecyl ester	8.89	0.23	$C_{28}H_{56}O_2$	424	Ester
3	4''-Dehydroxy-2'',3',3'',4',5,6'',7-heptaO-methylisoorientin	8.89	0.23	$C_{28}H_{34}O_{11}$	546	Flavonoid glycoside
4	Pyrrolidine, 1-(1,6-dioxooctadecyl)	9.19	0.42	$C_{22}H_{41}NO_2$	351	Pyrrolidine
5	*tert*-Hexadecanethiol	10.50	0.63	$C_{16}H_{34}O$	242	Alkanethiol
6	2-Hexyl-1-octanol	11.21	1.36	$C_{14}H_{30}O$	214	Alkyl alcohol
7	Oxiraneoctanoic acid, 3-octyl-, methyl ester	11.47	0.34	$C_{19}H_{36}O_3$	312	Oxriane (epoxide)
8	2H-Tetrahydropyran, 2-[(1-ethenyl-1,5-dimethylhex-4-enyl)oxy]	13.06	5.50	$C_{16}H_{30}O_4$	286	Alkoxoide

Table S4. Newly identified compounds of *B. paralicheniformis* (PK04) (***Continued***).

S. No	Compound name	RT (Min)	Area %	Molecular formula	Molecular weight	Chemical origin
9	Docosane, 9-butyl	14.18	5.25	$C_{26}H_{54}$	366	Alkane
10	1-Diphenylsilyloxytetradecane	15.22	6.88	$C_{26}H_{40}OSi$	396	Organosilicon alakne
11	9H-Pyrrolo[1',2':2,3]isoindolo[4,5,6-cd] indol-9-one	16.18	3.51	$C_{20}H_{20}N_2O_3$	336	Pyrroloisoindole
12	Ethaneperoxoic acid, 1-cyano-1-[2-(2-phenyl-1,3-dioxolan-2- yl)ethyl]pentyl ester	17.67	4.73	$C_{19}H_{25}NO_5$	347	Peroxy acid
13	Vitamin A palmitate	17.95	2.45	$C_{36}H_{60}O_2$	524	Vitamin
14	Emetan, 1',2'-didehydro-6',7',10,11-tetramethoxy	18.24	1.77	$C_{29}H_{38}N_2O_4$	478	Alkaloid
15	Palmitic anhydride	18.79	22.75	$C_{32}H_{62}O_3$	494	Anhydride
16	Oxiraneoctanoic acid, 3-octyl-, methyl ester	11.47	0.34	$C_{19}H_{36}O_3$	312	Epoxides

Table S4. Newly identified compounds of *B. paralicheniformis* (PK04) (***Continued***).

S. No	Compound name	RT (Min)	Area %	Molecular formula	Molecular weight (g/mol)	Chemical origin
17	Gibbane-1,10-dicarboxylic acid, 2,3-epoxy-4a,7-dihydroxy-1-methyl-8-m ethylene-, 1,4a-lactone, 10-methyl ester, (1à,2á,3á,4aà,4bà,10á)	20.23	1.62	$C_{20}H_{24}O_6$	360	Gibbane-type sesquiterpene
18	(5á,13à) 3à-Methoxy-3á,19-epoxyandrost-8-ene7à,17á-diol, 4,4-dimethyl	20.68	0.54	$C_{22}H_{34}O_4$	362	Steriod
19	Dasycarpidan-1-methanol, acetate (ester)	21.42	3.34	$C_{20}H_{26}N_2O_2$	326	Sesquiterpene ester
20	Phthalic acid, 6-ethyloct-3-yl 2-ethylhexyl ester	22.27	8.35	$C_{26}H_{42}O_4$	418	Ester
21	Olean-12-ene-3,15,16,21,22,28-hexol, (3á,15à,16à,21á,22à)	23.02	0.84	$C_{30}H_{50}O_6$	506	Triterpeniod
22	2,6,10,14,18,22-Tetracosahexaene, 2,6,10,15,19,23-hexamethyl-, (all-E)	23.59	5.03	$C_{30}H_{50}$	410	Careoteniod

Table S4. Newly identified compounds of *B. paralicheniformis* (PK04) (***Continued***).

S. No	Compound name	RT (Min)	Area %	Molecular formula	Molecular weight (g/mol)	Chemical origin
23	(22S)-21-Acetoxy-6à,11á-dihydroxy-16 à,17à-propylmethylenedioxy pregna-1,4-diene-3,20-dione	23.96	0.61	$C_{27}H_{36}O_8$	488	Steriod
24	Corynan-17-ol, 18,19-didehydro-10-methoxy-, acetate (ester)	24.57	1.43	$C_{22}H_{28}N_2O_3$	368	Alkaloid ester
25	7aH-Cyclopenta[a]cyclopropa[f]cyclou ndecene-2,4,7,7a,10,11-hexol, 1,1a,2,3,4,4a,5,6,7,10,11,11a-dodecahy dro-1,1,3,6,9-pentamethyl-, 2,4,7,10,11-pentaacetate	25.20	1.26	$C_{30}H_{44}O_{11}$	580	Polycyclic polyol ester
26	9,12,15-Octadecatrienoic acid, 2,3-bis[(trimethylsilyl)oxy]propyl ester, (Z, Z, Z)	25.65	2.12	$C_{27}H_{52}O_4Si_2$	496	Silylated ester

Table S4. Newly identified compounds of *B. paralicheniformis* (PK04) *(**Continued**).*

S. No	Compound name	RT (Min)	Area %	Molecular formula	Molecular weight (g/mol)	Chemical origin
27	1*H*-Cyclopropa[3,4]benz[1,2-e]azulene-4a,5,7b,9,9a(1aH)-pentol, 3-[(acetyloxy)methyl]-1b,4,5,7a,8,9-hexahydro-1,1,6,8-tetramethyl-, 5,9,9a-triacetate	26.02	4.14	$C_{28}H_{38}O_{10}$	534	Polycyclic alcohol ester
28	18,19-Secoyohimban-19-oic acid, 16,17,20,21-tetradehydro-16-(hydroxymethyl)-, methyl ester, (15á,16E)	26.90	1.06	$C_{21}H_{24}N_2O_3$	352	Secoyohimban-type alkaloid

Table S5. Pharmacokinetic properties of *B. paralicheniformis* (PK04) isolate.

S. No	Compound name	Flexibility	tPSA (Polarity) (Å²)	Bioavailability (F)	XlogP3 (Lipophilicity)	GI	BBB	Solubility	LogK_p (cm/s)
1	Phenanthro[1,2-c][1,2,5]selenadiazoe	0	25.78	0.55	2.86	High	Yes	Moderately soluble	-6.00
2	Decanoic acid, octadecyl ester	26	26.30	0.55	13.01	Low	No	Poorly soluble	0.35
3	4"-Dehydroxy-2",3',3",4',5,6",7-hepta O-methylisoorientin	6	165.89	0.55	2.27	Low	No	Moderately soluble	-8.02
4	Pyrrolidine, 1-(1,6-dioxooctadecyl)	16	40.54	0.55	6.16	High	Yes	Moderately soluble	-4.07
5	*tert*-hexadecanethiol	13	20.23	0.55	6.98	High	Yes	Moderately soluble	-2.82

Table S5. Pharmacokinetic properties of *B. paralicheniformis* (PK04) isolate *(**Continued**)*.

S. No	Compound name	Flexibility	tPSA (Polarity) (Å²)	Bioavailability (F)	XlogP3 (Lipophilicity)	GI	BBB	Solubility	LogK_p (cm/s)
6	2-Hexyl-1-octanol	11	20.23	0.55	5.92	High	Yes	Moderately soluble	-3.40
7	Oxiraneoctanoic acid, 3-octyl-, methyl ester	16	38.83	0.55	7.13	High	Yes	Moderately soluble	-3.14
8	2*H*-Tetrahydropyran, 2-[(1-ethenyl-1,5-dimethylhex-4-enyl)ox y]	17	43.37	0.55	6.34	High	Yes	Moderately soluble	-3.70
9	Docosane, 9-butyl	22	0.001	0.55	14.01	Low	No	Poorly Soluble	1.41
10	1-Diphenylsilyloxytetradecane	15	9.23	0.55	8.80	Low	No	Poorly Soluble	-1.96
11	9H-Pyrrolo[1',2':2,3]isoindolo [4,5,6-cd] indol-9-one	1	73.40	0.55	2.23	High	Yes	Moderately soluble	-6.77

Table S5. Pharmacokinetic properties of *B. paralicheniformis* (PK04) isolate ***(Continued)***.

S. No	Compound name	Flexibility	tPSA (Polarity) (Å²)	Bioavaila-bility	XlogP3 (Lipophi-licity)	GI	BBB	Solubility	LogK_p (cm/s)
12	Ethaneperoxoic acid, 1-cyano-1-[2-(2-phenyl-1,3-dioxolan-2- yl)ethyl]pentyl ester	10	77.78	0.55	3.24	High	Yes	Moderately soluble	-6.12
13	Vitamin A palmitate	21	26.30	0.55	13.58	Low	No	Poorly Soluble	0.14
14	Emetan, 1',2'-didehydro-6',7',10,11-tetramethoxy	7	52.19	0.55	5.30	High	Yes	Poorly Soluble	-5.90
15	Palmitic anhydride	30	43.37	0.55	14.55	Low	No	Insoluble	1.01
16	Oxiraneoctanoic acid, 3-octyl-, methyl ester	16	38.83	0.55	7.13	High	Yes	Moderately soluble	-3.14
17	Gibbane-1,10-dicarboxylic acid, 2,3-epoxy-4a,7-dihydroxy-1-methyl-8-methylene-, 1,4a-lactone, 10-methyl ester, (1à,2á,3á,4aà,4bà,10á)	1	83.83	0.56	1.68	High	No	Soluble	-7.13

Table S5. Pharmacokinetic properties of *B. paralicheniformis* (PK04) isolate *(**Continued**)*.

S. No	Compound name	Flexibility	tPSA (Polarity)	Bioavailability (F)	XlogP3 (Lipophilicity)	GI	BBB	Solubility	LogK_p (cm/s)
18	(5á,13à) 3à-Methoxy-3á,19-epoxyandrost-8-ene7à,17á-diol, 4,4-dimethyl	16	52.60	0.55	7.02	High	No	Moderately soluble	-3.53
19	Dasycarpidan-1-methanol, acetate (ester)	4	45.33	0.55	3.21	High	Yes	Soluble	-6.01
20	Phthalic acid, 6-ethyloct-3-yl 2-ethylhexyl ester	17	52.60	0.55	8.41	Low	No	Poorly Soluble	-2.88
21	Olean-12-ene-3,15,16,21,22,28-hexol, (3á,15à,16à,21á,22à)	2	121.38	0.55	3.97	High	No	Moderately soluble	-6.57

Table S5. Pharmacokinetic properties of *B. paralicheniformis* (PK04) isolate *(**Continued**)*.

22	2,6,10,14,18,22-Tetracosahexaene, 2,6,10,15,19,23-hexamethyl-, (all-E)	15	0.001	0.55	7.84	Low	No	Moderately soluble	-2.73
23	(22S)-21-Acetoxy-6à,11á-dihydroxy-16 à,17à-propylmethylenedioxy pregna-1,4- diene-3,20-dione	9	116.20	0.55	4.22	High	No	Moderately soluble	-6.28
24	Corynan-17-ol, 18,19-didehydro-10-methoxy-, acetate (ester)	7	58.64	0.55	3.70	High	Yes	Moderately soluble	-5.92

Table S5. Pharmacokinetic properties of *B. paralicheniformis* (PK04) isolate *(**Continued**)*.

S. No	Compound name	Flexibility	TPSA (Polarity) (Å²)	Bioavailability (F)	XlogP3 (Lipophilicity)	GI	BBB	Solubility	LogK_p (cm/s)
25	7aH-Cyclopenta[a]cyclopropa[f]cyclou ndecene-2,4,7,7a,10,11-hexol, 1,1a,2,3,4,4a,5,6,7,10,11,11a-dodecahy dro-1,1,3,6,9-pentamethyl-, 2,4,7,10,11-pentaacetate	10	151.73	0.55	2.75	Low	No	Moderately soluble	-7.89
26	9,12,15-Octadecatrienoic acid, 2,3-bis[(trimethylsilyl)oxy]propyl ester, (Z,Z,Z)	21	44.76	0.55	8.84	Low	No	Poorly Soluble	3.05

Table S5. Pharmacokinetic properties of *B. paralicheniformis* (PK04) isolate *(**Continued**)*.

S. No	Compound name	Flexibility	TPSA (Polarity) (Å²)	Bioavailability (F)	XlogP3 (Lipophilicity)	GI	BBB	Solubility	LogK_p (cm/s)
27	1*H*-Cyclopropa[3,4]benz[1,2-e]azulene -4a,5,7b,9,9a(1aH)-pentol, 3-[(acetyloxy)methyl]-1b,4,5,7a,8,9-hex ahydro-1,1,6,8-tetramethyl-, 5,9,9a-triacetate	8	142.50	0.55	2.21	Low	No	Moderately soluble	7.99
28	18,19-Secoyohimban-19-oic acid, 16,17,20,21-tetradehydro-16-(hydroxy methyl)-, methyl ester, (15á,16E)	4	45.33	0.55	4.11	High	Yes	Moderately soluble	-5.46

Footnotes: Skin permeability coefficient (LogK_p), Topological Polar Surface Area (TPSA), Blood–Brain Barrier (BBB), Gastrointestinal (GI), Retention time (RT)

A.

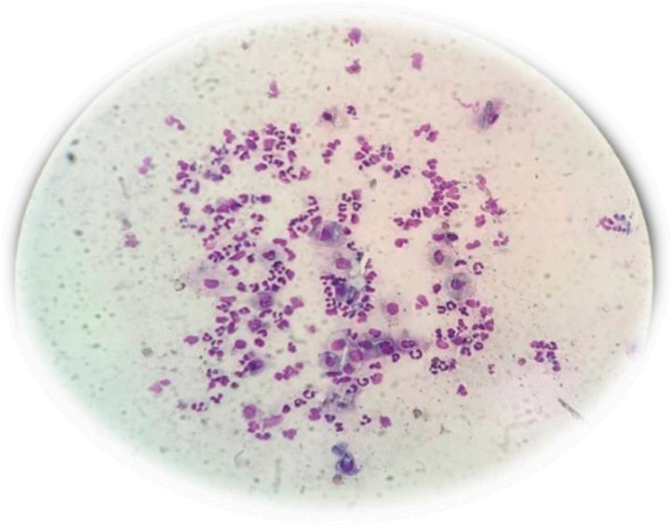

Gram-Positive

B.

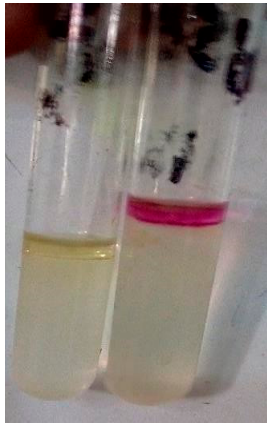

Indole Test

C.

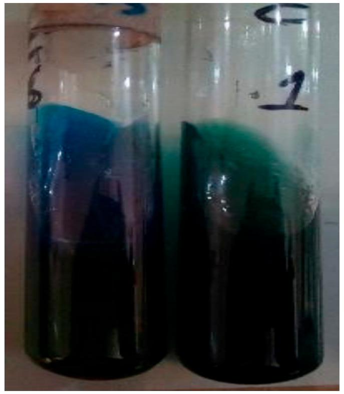

Citrate Test

D.

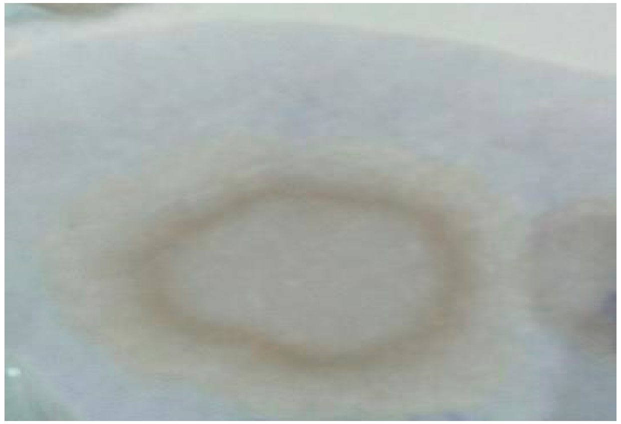

Oxidase Test

E.

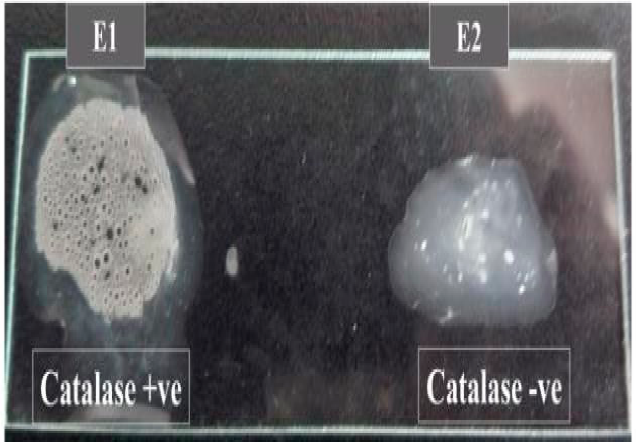

Catalase Test

F.

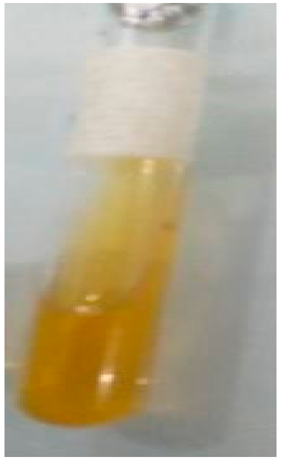

TSI Test

G.

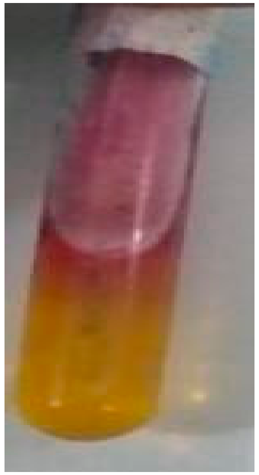

Urease Test

Figure S1. Culture and biochemical identification of bacterial isolates. **A**: Gram-positive, **B**: Indole, **C**: Citrate, **D**: Oxidase, **E**: Catalase, (E_1: Positive, E_2: Negative), **F:** TSI & **G:** Urease Test.

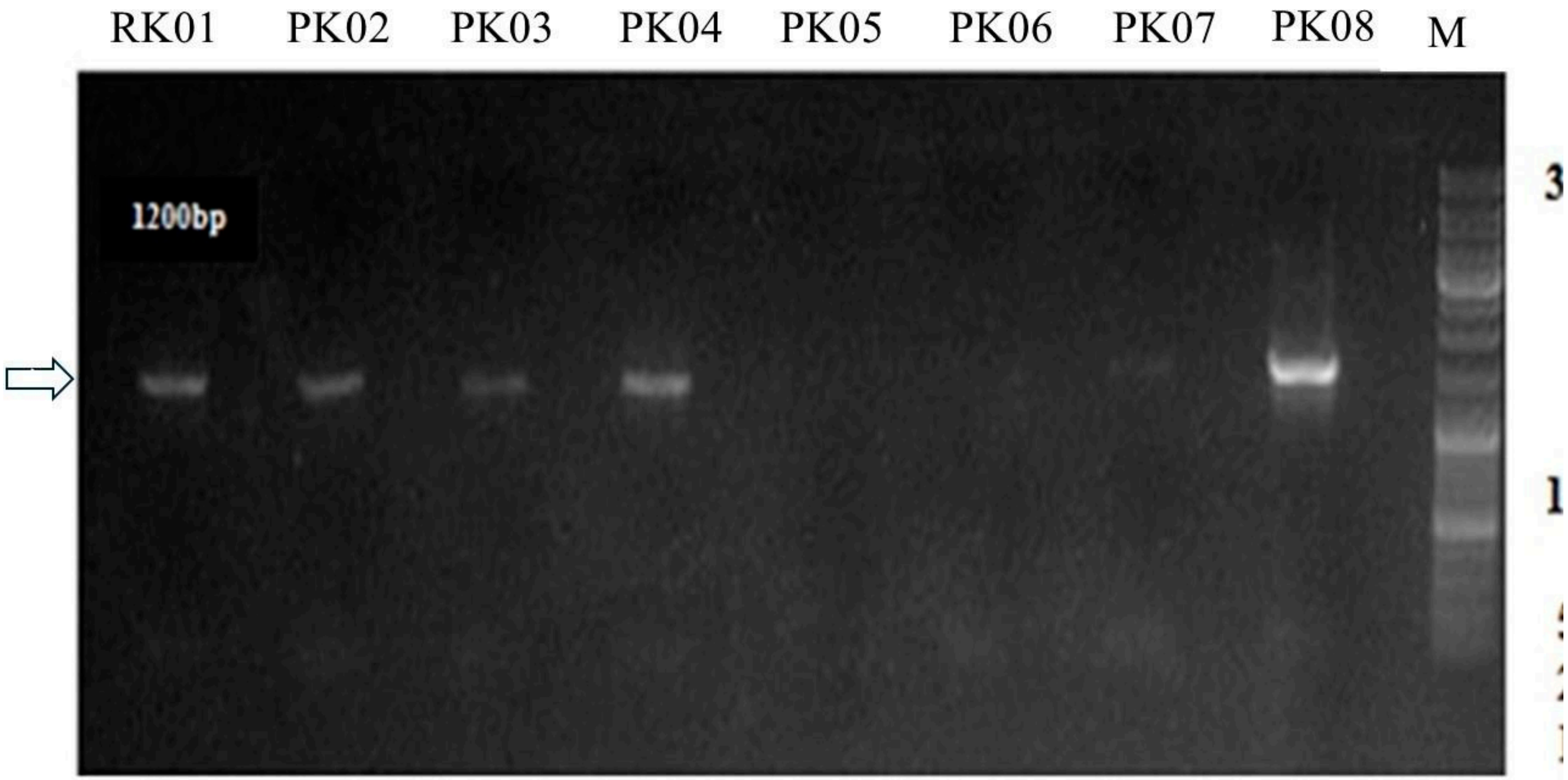

Figure S2. Amplification of 16S rRNA gene of soil bacteria: M = Marker, RK01, PK02, PK03, PK04, PK05, PK06, PK07, PK08 = Soil Isolates, bp = 16S rRNA gene bands. A preliminary screening of isolated species was performed, and based on bioactivity results, two bacterial species (RK01 and PK04) were selected for this study.

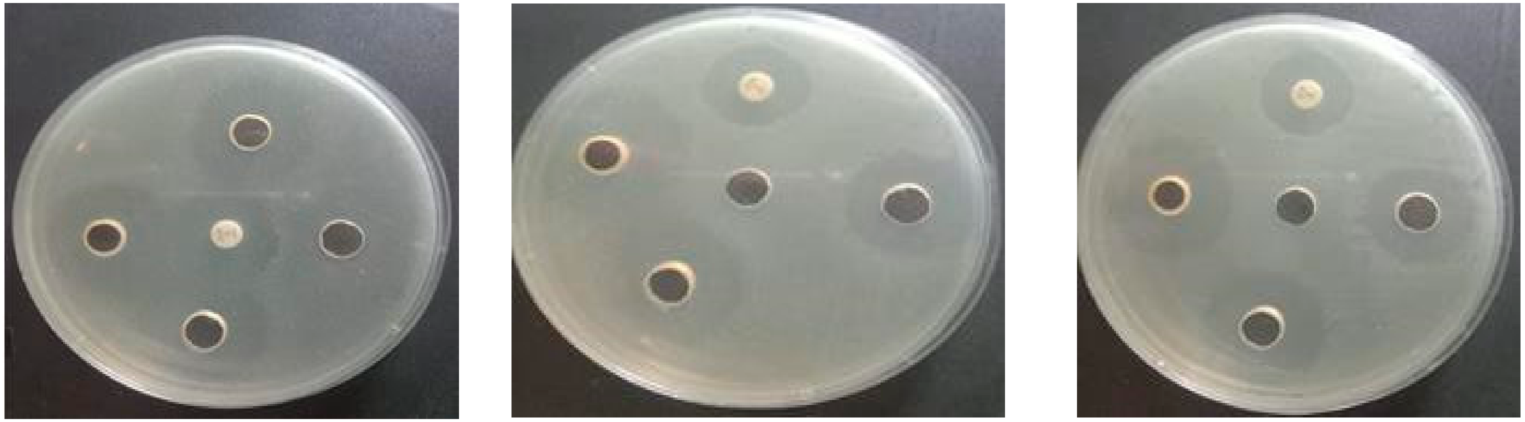

a b c

Figure S3. Antibacterial Activity against: (a) *Shigella flexneri, (b) Salmonella typhi, (c) E. coli.* A single concentration of 60 µL was employed; the antibiotic shows a positive control, whereas the well that has no zone of inhibition shows the negative control (DMSO). The antibiotic applied to the plate served as a positive control, while the well without a zone of inhibition represents the negative control (DMSO).
